# Microscopical Examination of Suspected New Growths

**Published:** 1903-08-15

**Authors:** 


### Hospital Clinics and Medical Progress.

### MICROSCOPICAL EXAMINATION OF SUSPECTED NEW GROWTHS.

Where malignant disease is suspected it is advisable and often necessary to have the diagnosis established before deciding to undertake an operation extensive enough for the removal of the whole disease. Especially is this confirmation important in the case of a doubtful wart or ulcer in the mouth, *i.e.* tongue, cheek, or palate, or of the cervix uteri. The selection of a suitable piece of the suspected material is very important. When an ulcer is to be examined, a wedge-shaped piece should be taken from the edge so that the apex of the wedge includes some of the floor of the ulcer, while the broad base consists of tissue beyond the ulcerating margin; the piece should also extend well down into the deeper tissues. A portion of the margin of an ulcer cut out in this way will, on microscopic examination, make it possible for an opinion to be formed as to its malignancy or otherwise, with a good deal of certainty, for the two most important points will be shown:—(1) The nature of the growth at the edge, for instance a downgrowth of surface epithelium in the case of an epithelioma; and (2) the infiltration or non-involvement of the deeper structures. It often happens that there is a more or less warty condition about the edge of a simple chronic ulcer, the innocency of which can only be established by examining a piece which includes the surrounding healthy tissue; the extent and depth of the piece taken is then of much importance. The great advantage obtained by the microscopic examination of a suspected malignant ulcer is, that if the lesion turn out to be malignant, it justifies the operator making a wide sweep including at least $\frac{3}{8}$ inch, and more if possible, of apparently unaffected tissue beyond the edge of the growth; and, on the other hand, if the microscope show that it is in what is called a precancerous condition, a less amount of surrounding healthy tissue need be sacrificed.

Another class of cases in which a preliminary examination is of service is that of tumours of the breast. Here the question of malignancy involves consideration of the removal of axillary glands and an extensive portion of skin, in addition to the breast itself. A large number of mammary tumours can be diagnosed with comparative certainty after clinical examination alone; there are, however, some cases in which it is not so easy to pronounce definitely that a certain breast is the seat of undoubted malignant disease or not. In these a preliminary examination of a portion of the suspicious growth is essential. In a doubtful case the lump should be cut down upon: a surprise not infrequently awaits the operator at this stage—a cyst, with tense and thick wall, embedded in the breast; or a thickened and indurated mass of breast tissue, or a definite lump of hard carcinoma may be revealed. These several conditions may be quite evident, but sometimes that which looks like chronic mastitis turns out to be malignant disease, or new growth may be commencing outside the wall of a cyst. It is therefore a great advantage to have these points settled if possible at once. This can be done on the spot, before proceeding further with the operation, according to the following plan:—A piece of the suspicious part is removed and laid in some gum on the metal disc of a freezing microtome. It is then frozen, sections are cut, and one or two of these are floated out in some clean water in a large glass dish. The dish should stand on a dark or black background to show up the sections, and the latter can be made to float out by dipping them first in methylated spirit and then placing them in the water, the currents set up by the spirit in the water cause them to rapidly unfold. A section is lifted out on an ordinary glass slide, the excess of fluid is drained off, and one or two drops of filtered Loeffler's methylene blue are dropped upon the spread out section, a cover glass is then immediately placed on the stain and section, excess of the stain is removed by pressing the slide between layers of filter paper and the specimen is ready for microscopic examination. This method has been used by the writer for some years, and has always been successful; the time taken is quite short, usually about five to ten minutes. The success of the proceeding depends upon having everything in readiness beforehand. The most satisfactory instrument for use is William's ether freezing microtome, which can be securely clamped to a table.

The confirmation of the presence of carcinoma would justify the immediate amputation of the whole breast, and removal of axillary glands. On the other hand, if only chronic mastitis were found the affected part only would need excision. The saving of skin over a breast affected with general fibro-cystic disease to the extent of requiring complete removal is worth considering. This method of rapid diagnosis at the time of operation is applicable to cases other than those of breast tumours. Suspicious ulcers about the mouth or cervix uteri, abdominal tumours, suspected malignant growths of the arm or leg; in fact there is no part of the body in which the immediate settlement of the nature of a particular tumour or ulcer may not be of the greatest importance.

If it is not convenient to cut sections at once by the above method, a piece of the tumour or the edge of the ulcer should be removed and placed in some hardening reagent such as Muller's fluid (sodium sulphate, 1 part; potassium bichromate, $2\frac{1}{4}$ parts; and tap water, 100 parts), methylated spirit, 50 per cent., or formalin, 10 per cent. solution. Of these Muller's fluid is the best. The specimen should be sent to a competent pathologist for sections to be prepared and a diagnosis made. Small bottles with an ordinary cork stopper and holding about an ounce of fluid, and packed in tins or cardboard boxes lined with corrugated thick brown paper, are admirably suited for transit of specimens through the post. A simpler plan is to wrap the specimen in gutta percha tissue and seal it up with turpentine or chloroform, and then to wrap a piece of waterproof outside this and post in an envelope with letter of instruction. A bottle is safer however for a delicate specimen. The particulars sent with the specimen to the pathologist are important. Medical men in the hurry of general practice have very little time for letter writing, but it is absolutely essential if the proper diagnosis of a microscopic section is to be made, for the pathologist to have certain clinical facts placed before him. It happens over and over again that a specimen is received for microscopic examination accompanied by a brief note saying, "Would you kindly report on the enclosed lump which I removed to-day?" Another favourite way is for the dispenser to write, "Dr. A. would be obliged for an early report on the enclosed specimen." This is obviously unfair both to the pathologist and to the patient. The details given should include:—(1) The age and sex of the patient, (2) the situation of growth, (3) the length of time it has been noticed and the rate of growth recently, (4) the size and consistency of the tumour, and whether it was infiltrating or encapsuled, (5) whether or not there were any enlarged glands in its neighbourhood, (6) the opinion of surgeon himself as to whether he thinks it malignant or not from a clinical standpoint.

All this may seem very elementary, but it is a great help to the pathologist, for the microscope, although of the greatest service, is not all powerful. Many specimens offer no difficulties, and can be diagnosed almost immediately on looking down the microscope, without even a word of clinical history; but there are others in which advantage has to be taken of facts in the history of the case if a correct opinion is to be arrived at. Even then there are still a few cases in which it is quite impossible to arrive at a definite conclusion.

After removal of a growth, apart from the natural interest of seeing the microscopic structure of the tumour which has been removed, and the clinical features of which are familiar, the question of prognosis arises. Sometimes the pathological investigation proves a specimen to be of a nature wholly different from that suggested at the time of removal; for instance: a young woman presented herself with a small nodule in the skin of the arm which had been there a year or two, and had been shaved off with a knife once under the impression that the nodule was an ordinary wart; the lump reappeared, and as it was unsightly the patient wished to get rid of it. The nodule was therefore cut out, and, purely as a matter of curiosity, sections were made, with the result that it was found to be microscopically a round-celled sarcoma. A second case illustrates the same point, this time a "nævoid growth" was removed from the skin of the arm of a young man; the specimen was not examined microscopically. A month later the man had a similar though larger mass removed from the same situation, this piece was examined microscopically, and turned out to be a round-celled sarcoma, and for this reason the arm was subsequently amputated close to the shoulder joint; the growth was found to be infiltrating the muscles of the limb.

Cases like the above are often met with, and illustrate the great value of a microscopical examination of every tumour removed in making a definite diagnosis.

